# Attentional dynamics during free picture viewing: Evidence from oculomotor behavior and electrocortical activity

**DOI:** 10.3389/fnsys.2013.00017

**Published:** 2013-06-04

**Authors:** Thomas Fischer, Sven-Thomas Graupner, Boris M. Velichkovsky, Sebastian Pannasch

**Affiliations:** ^1^Engineering Psychology and Applied Cognitive Research, Department of Psychology, Technische Universitaet DresdenGermany; ^2^Department of Cognitive Studies, Kurchatov InstituteMoscow, Russia; ^3^Brain Research Unit and MEG Core, O.V. Lounasmaa Laboratory, School of Science, Aalto UniversityEspoo, Finland

**Keywords:** eye fixation-related potentials, saccadic eye movements, top-down attention, bottom-up attention, sustained attention, EEG

## Abstract

Most empirical evidence on attentional control is based on brief presentations of rather abstract stimuli. Results revealed indications for a dynamic interplay between bottom-up and top-down attentional mechanisms. Here we used a more naturalistic task to examine temporal signatures of attentional mechanisms on fine and coarse time scales. Subjects had to inspect digitized copies of 60 paintings, each shown for 40 s. We simultaneously measured oculomotor behavior and electrophysiological correlates of brain activity to compare early and late intervals (1) of inspection time of each picture (picture viewing) and (2) of the full experiment (time on task). For picture viewing, we found an increase in fixation duration and a decrease of saccadic amplitude while these parameters did not change with time on task. Furthermore, early in picture viewing we observed higher spatial and temporal similarity of gaze behavior. Analyzing electrical brain activity revealed changes in three components (C1, N1 and P2) of the eye fixation-related potential (EFRP); during picture viewing; no variation was obtained for the power in the frontal beta- and in the theta activity. Time on task analyses demonstrated no effects on the EFRP amplitudes but an increase of power in the frontal theta and beta band activity. Thus, behavioral and electrophysiological measures similarly show characteristic changes during picture viewing, indicating a shifting balance of its underlying (bottom-up and top-down) attentional mechanisms. Time on task also modulated top-down attention but probably represents a different attentional mechanism.

## Introduction

When exploring our visual environment, the sampling of information is based on sequences of single eye fixations guided by visual attention. The concept of visual attention describes how the attentional focus moves (e.g., Peelen and Kastner, 2011) and how the focused information is processed (e.g., Hillyard et al., 1998). A well-established approach about the control of attention characterizes two distinct modes of information selection (James, 1890; Kinchla, 1992): In the *bottom-up* mode (stimulus-driven or exogenous control), information selection is guided by low-level visual features such as physical and biological saliencies (Itti and Koch, 2001; Ohman et al., 2001) or is captured by transient changes such as stimulus onset or motion (Egeth and Yantis, 1997; Peters et al., 2005). In the *top-down* mode (goal-driven or endogenous control) information selection is guided by internal goals, knowledge, or task instructions (Egeth and Yantis, 1997). While there is agreement on the existence of such two attentional modes, there is a lack of consensus on the interaction between them, particularly about the relative timing and the neural mechanisms of their activity (Chun et al., 2011).

Although theoretical concepts often propose simultaneous activity of both modes of attentional control (Egeth and Yantis, 1997; Itti and Koch, 2001; Corbetta et al., 2008), empirical findings often reveal differences in the engagement of both mechanisms at least within short time periods: Immediately after the onset of a new stimulus, bottom-up control is dominating before top-down control becomes more influential over time (Van der Stigchel et al., 2009; Hickey et al., 2010). Other authors reported an immediate influence of top-down factors, such as task demands (Einhäuser et al., 2008). Throughout the time course of attentional deployment it is furthermore unclear if the influence of bottom-up control decreases (Parkhurst et al., 2002) or if it remains stable but additional top-down regulation comes into play (Tatler et al., 2005). The analysis of psychophysiological indicators of the temporal interaction so far has mainly been conducted on the scale of milliseconds and seconds (Theeuwes, 2010). Examining behavioral and psychophysiological indicators of attention during more natural tasks would allow generalizing previous results.

At the behavioral level, indications have been found that attention changes over longer time intervals during naturalistic viewing: Eye movement analyses revealed that regions of high saliency, i.e., objects that clearly stand out from the background, are fixated earlier than less salient objects if no particular instruction is provided (Underwood and Foulsham, 2006; Underwood et al., 2006). This has been interpreted as an early dominance of bottom-up processing, where our gaze is captured by low level features of high saliency and was confirmed by higher interindividual consistency of gaze locations early in scene inspection (Parkhurst et al., 2002; Tatler et al., 2005; Masciocchi et al., 2009). In contrast, the interindividual consistency decreased later during inspection which was attributed to a stronger influence of top-down regulation on the viewing behavior due to the individually gathered knowledge (Velichkovsky, 2002; Henderson, 2003). According to Tatler et al. (2011), there are problems with this interpretation. On one hand, natural scenes often have a small but reliable bias for high salient objects being rather located in the center; on the other hand, there is a tendency that central regions of an image are fixated more often early in scene inspection. Such a “central fixation bias” may reflect a general tendency for observers to fixate near the center of scenes, irrespective of saliency (Tatler et al., 2005, p. 650) and thus may be unrelated to bottom-up control of attention.

Apart from gaze locations, changes in fixation durations and saccadic amplitudes during longer inspection times were reported for naturalistic viewing. Within 2 s after the image onset, fixations were shorter and saccades were larger compared to later stages of scene exploration (Unema et al., 2005; Pannasch et al., 2008). Recently, it was found that disrupting top-down guidance by scrambling the picture content subsequent fixations became shorter while the saccadic amplitudes increased (Foulsham et al., 2011).

In contrast to the observations of gaze behavior, less is known about the dynamics of psychophysiological indicators (e.g., EEG) during longer intervals (>2 s) of naturalistic viewing. One reason is probably that conventional analyses cannot adequately take into account the appearance of sequential eye movements. Here, the analysis of EEG epochs time-locked to onsets of eye fixations (i.e., eye fixation-related potentials, EFRP) is necessary. Using this method revealed similar results as in more traditional experiments where cortical responses are locked to a sudden stimulus change (e.g., Yagi, 1979; Graupner et al., 2007, 2011; Rama and Baccino, 2010). The neuronal sources of EFRPs in scene viewing are mainly distributed across occipital and parietal regions and are primarily characterized by the components P1, N1, and P2. Recent evidence also suggests the existence of an early C1 component in the EFRP during picture perception (see Figure 3 in Graupner et al., 2011).

Early components such as C1, P1, N1, and P2 are usually assumed to be controlled by physical stimulus properties (Hopfinger and Ries, 2005). In contrast, later components such as N2, P3, and N4 are rather thought to reflect top-down processing (see e.g., Donchin et al., 1978). While this distinction of the components seems appealing in the context of describing attentional mechanisms, it is presumably too simple. Top-down regulation, for instance, has also been found to influence C1, P1, N1, and P2 (Johannes et al., 1995; Freunberger et al., 2007; Rauss et al., 2009, 2011; Wykowska and Schubo, 2010). Specifically, for the N1, influences of working memory (WM) load were found. During a visual selection paradigm the N1 was smaller when WM demands were high (Rose et al., 2005). Similar influences were also found in WM paradigms with auditory evoked N1 components (Conley et al., 1999; Golob and Starr, 2004) and in a spatial WM paradigm (Rader et al., 2008). Furthermore, de Fockert et al. (2001) found a strong connection between WM and visual selective attention, demonstrating that WM can reduce visual distraction due to the prioritization of relevant information. The few investigations that analyzed the functional aspects of the P2 component demonstrated its association with visual selective attention and WM (Freunberger et al., 2007). When irrelevant stimuli were presented before target presentation the P2 increased as function of distraction (Vierck and Miller, 2009).

Even for C1—the earliest component of the ERFP-complex—results suggest a susceptibility to top-down modulation (Rauss et al., 2011). Nevertheless, the majority of evidence has found bottom-up related influences on C1 (Khoe et al., 2005; Stolarova et al., 2006), in particular by effects of saliency (Zhang et al., 2012). Therefore, we expect that large C1 amplitudes during naturalistic viewing should be associated with stronger bottom-up control. The C1 amplitude should become smaller when bottom-up influences are less important (i.e., later during inspection). With increasing inspection time, we not only postulate a diminishing impact of bottom-up attention but also a shift toward a stronger top-down controlled mode of attention. Such stronger top-down regulation could be triggered for instance by increased demands on WM and selective attention that might result in decreased N1 and increased P2 amplitudes.

Another important function of top-down control describes the ability to maintain an adequate level of internal arousal to fulfill demands of an ongoing task over longer periods. This ability is associated with the concept of sustained attention and characterized as the effort to compensate the negative outcomes of decreasing arousal, known and well documented as increasing subjective sleepiness and fatigue with time on task (Parasuraman et al., 1998; Lorist et al., 2000).

Demands on sustained attention have been found to correlate with the amount of power in frontal theta and beta frequency band of the EEG (Arruda et al., 1999; Sauseng et al., 2007). Therefore, we expect increased power in theta and beta frequency bands during later phases of the experiment. So far it is not known to what extent demands on sustained attention are required to maintain performance in shorter tasks (<1 min). We expect to contribute to this question by comparing frequency power between early and late periods of image inspection.

To examine attentional mechanisms on a larger time scale, our subjects freely explored paintings for a period of 40 s. Paintings are considered as “maximal memory stores” (Leyton, 2006, p. 2). Their inspection requires active exploration in combination with time-consuming accumulation of knowledge which corresponds well with demands on attention in everyday activities. During our experiment we predicted changes at two different time scales. Firstly, we expect changes throughout the 40 s of inspection of each picture (henceforth picture viewing) indicating variations in the balance of bottom-up and top-down attention. Secondly, we presume changes throughout the time course of the whole experiment (henceforth time on task). Such variation should indicate various demands on sustained attention. To best of our knowledge, behavioral and psychophysiological correlates of bottom-up, top-down and sustained attention have never been investigated using such a naturalistic task.

## Materials and methods

### Subjects

Twenty-seven healthy volunteers (5 males, mean age 23.5, age range 18–35) participated in the experiment. All subjects had normal or corrected to normal vision and received either course credit or monetary reward for their participation in the study that was conducted in conformity with the declaration of Helsinki and approved by the Ethics Committee of the Technische Universitaet Dresden. Written informed consent was obtained from all participants.

### Apparatus

Participants were seated in a dimly illuminated, sound-attenuated room. Eye movements were recorded monocularly at 500 Hz using the EyeLink 1000 infrared eye tracking system (SR Research, Ontario, Canada), operated in the remote mode. The system allows continued eye movement recordings with a spatial resolution below. 0.01° and a spatial accuracy of better than 0.5°. The distance between the eye-tracking device and the subjects' eye was always about 60 cm. The eye tracker and the experimental procedure were controlled using the Experiment Builder software (SR Research, Ontario, Canada). Saccades and fixations were defined using the saccade detection algorithm supplied by SR Research: Saccades were identified by deflections in eye position in excess of 0.1°, with a minimum velocity of 30° s^−1^ and a minimum acceleration of 8000° s^−2^, maintained for at least 4 ms.

EEG activity was recorded using a Brain Amp DC-amplifier. Sixty-four electrodes were placed according to the standard 10/10 system. Data were collected in a shielded room with 500 Hz sampling rate and a high pass filter at 0.1 Hz. Both mastoids were used as reference and earlobes served as ground. All electrode impedances were kept below 5 kΩ.

We furthermore employed the Short Questionnaire for Current Strain (KAB; Mueller and Basler, 1992) to measure current subjective strain. The KAB is a self-report questionnaire including eight pairs of adjectives on a 6-point Likert-type rating scale describing opposite endpoints of different strain dimensions (e.g., stressed vs. relaxed; languid vs. fresh). The Stanford Sleepiness Scale (SSS; Herscovitch and Broughton, 1981) quantifies sleepiness based on seven bipolar items and was used to record changes in fatigue over the course of the experiment.

### Stimuli and procedure

Sixty digitized copies of representational paintings by different 16th and 17th century European artists were presented in random order. As there was variation in the format of the original paintings, they were proportionately rescaled to fit either the width or height of the display device resolution (1024 × 768 pixels). Stimuli were presented using a JVC DLA G11 video projector at a refresh rate of 60 Hz. The size of the projection screen was about 110 by 80 cm; viewed from a distance of 180 cm, the screen subtended a visual angle of 33° horizontally and 25° vertically. Before signing the consent form, participants were informed that the purpose of the study was to investigate eye movement behavior and brain activity in perception of art. They were asked to freely inspect and enjoy the images as they would do in an art gallery. An initial 9-point calibration and validation was performed before the start of the first trial and after the break; calibration was checked prior to each trial. A trial started with an 8 s presentation of a random pixel image—created from the subsequently shown image—followed by a central white fixation cross shown for 1.5–3 s. During the presence of the fixation cross, participants had to fixate it until the real image was shown for an inspection time of 40 s. After half of the trials, subjects were given a short break of 5 min. The total duration of the experiment was approximately 1 h. Prior and after the experimental session subjects had to complete both questionnaires, the KAB and the SSS.

### Data analysis

We employed two different analysis strategies to examine the behavioral and psychophysiological data. Possible short term changes during picture viewing were examined by dividing the 40-s viewing period in particular time intervals (for details see below). For the time on task investigation (i.e., examining changes on a larger time scale), we distinguished between early (first 20 images) and late (last 20 images) parts of the experiment.

#### Behavioral data

Gaze behavior was analyzed in terms of fixation duration, saccade amplitude and viewing similarity. We excluded fixations preceded or followed by blinks, fixations shorter than 120 ms, and those fixations during which the image onset and offset took place. To examine effects of 40 s of picture viewing the eye movement data was segmented into four 10-s bins per image.

For the analysis of fixation duration and saccade amplitude, we calculated the median value per subject for the respective time interval. Examination of viewing similarity is based on the chronological order of fixation locations and fixation durations. The analysis of viewing similarity employed the ScanMatch method (Cristino et al., 2010), using a 8 × 8 substitution matrix, dividing the screen in 64 sectors of 128 × 96 pixels. We used a gap penalty of “0” as it “benefits the global alignment of the sequences” (Cristino et al., 2010). For temporal binning, we applied a value of 325, since the median of all fixation durations was 326 ms. Thus, in the sequence a fixation of 325 ms was counted only once while a fixation of 650 ms was counted two times.

#### Psychophysiological data

To analyze the effect of picture viewing time on fixation related activity in the EEG we compared EFRPs from the first 10 s (early) to that from the remaining 30 s (late). Early and late EFRPs were matched by selecting fixations with durations of >300 ms and preceding saccade amplitudes of >3° from early and late time intervals. For each of the early fixations a gaze event from the late interval was selected based on two criteria: (1) the preceding saccade length belonged to the same quartile and (2) fixations were located at the same image region within a range of 3°. The same matching procedure was applied to study time on task effects, except for the gaze position criterion since congruency of the low-level visual features can hardly be achieved between the different stimuli of first and last 20 pictures. Hence, different sets of EFRPs were used for comparing early and late stages during picture viewing and for the analysis of time on task influences across the whole experiment.

For artifact rejection of the EEG, data were picture-wise epoched into 40-s segments. A blind source analysis (SOBI) was computed using the EEGLAB Matlab toolbox (Delorme and Makeig, 2004). The resulting components were visually inspected, to manually reject those components that were related to muscle or eye-ball activity. After artifact rejection the onsets of the selected fixations were used to create EFRP-segments. Subsequently, the EEG was segmented in epochs ranging from 200 ms before fixation onset to 500 ms afterwards. The −200 to −50 ms interval prior to fixation onset served for baseline correction. After preprocessing, an average of 99 (*SD* = 37.7) pairs of EFRPs per subject remained for the within picture comparison and an average 248 (*SD* = 68.3) pairs of EFRPs remained for the across picture comparison.

A parieto-occipital cluster, including the electrode positions PO3, POz, and PO4, was chosen to evaluate activity of the EFRP components. To define the EFRP components, we used the mean activity subsequent to the fixation onset with the following temporal boundaries: C1: 30–60 ms, P1: 90–120 ms, N1: 130–170 ms, and P2: 180–250 ms. For the analysis of activity in the frequency domain of the EFRPs, we calculated mean power of the theta (5–8 Hz) and beta (13–18 Hz) band for a fronto-central cluster, including Fpz, F3, Fz, F4, and FCz electrode sites. Multivariate analyses of variances (ANOVAs) were performed to separately evaluate the effects of picture viewing and time on task on the EFRP components (C1, P1, N1, P2) and on the frequency-band-power. Univariate statistics were performed to disentangle the specific effects. All steps of the EEG data processing were carried out using the Matlab toolbox EEGLAB (version 10) and all statistical analyses were performed with the SPSS 17.0 software package.

## Results

### Subjective data

Analysis of the SSS revealed increased sleepiness over time, *F*_(1, 24)_ = 23.7, *p* < 0.001. Self-reported sleepiness was significantly lower before (*M* = 2.08, *SD* = 0.76) than after the experiment (*M* = 3.04, *SD* = 0.94). Similarly, subjective strain as indicated by KAB values increased significantly, *F*_(1, 23)_ = 24.4, *p* < 0.001, from the start (*M* = 16.8, *SD* = 4.35) to the end of the experiment (*M* = 21.8, *SD* = 6.71).

### Behavioral data

**Figure 1:**
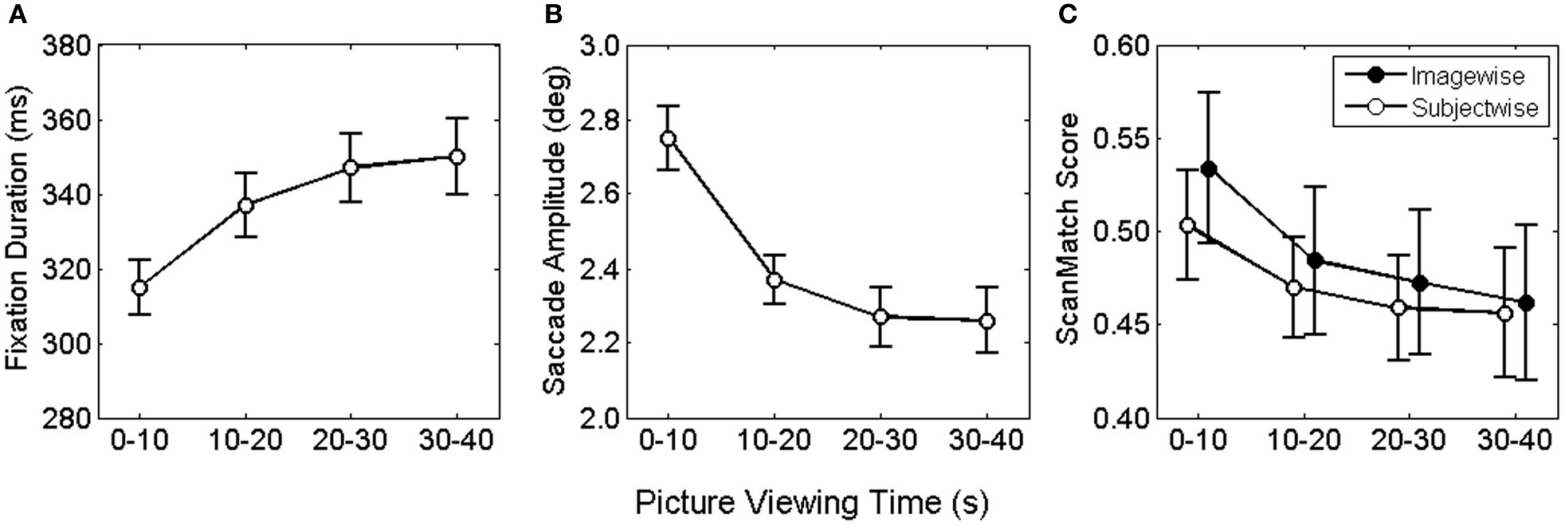
**Mean fixation duration (A) saccade amplitude (B) and viewing similarity (C) as function of viewing time.** Error bars depict the mean standard error.

Median fixation durations and saccade amplitudes were entered into two two-factorial repeated measures ANOVA with picture viewing (0–10, 10–20, 20–30, 30–40 s) and time on task (first vs. last 20 pictures) serving as within-subjects factor. For fixation durations, we found a significant main effect for picture viewing, *F*_(3, 78)_ = 23.9, *p* < 0.001. This effect was consistent across the whole experiment, as no influences of time on task and no interaction effect were observed, both *F* < 1.86. Figure 1A illustrates the asymptotic increase of fixation duration across the four bins of viewing time. Bonferroni corrected pairwise comparisons revealed a significant increase in fixation duration from the first to the second and from the second to the third time bin.

For saccade amplitude we also obtained a significant main effect for picture viewing, *F*_(3, 78)_ = 49.7, *p* < 0.001, but no influence of time on task and no interaction, both *F* < 1. As shown in Figure 1B saccadic amplitude decreased in an asymptotic fashion. Pairwise comparisons of viewing time confirmed the decrease only from the first to the second and form the second to the third bin.

Fixation locations and durations along the time course of exploration were used to examine viewing similarity imagewise and subjectwise. For the imagewise analysis, viewing sequences of all subjects for a particular painting were pairwise compared for each respective time bin. Each comparison produced a ScanMatch score (normalized between 0 and 1), indicating the similarity magnitude as distance from 0. The obtained ScanMatch scores for an image were averaged, resulting in one similarity index per painting. Equally, for the subjectwise analysis, viewing sequences of one subject for all paintings were pairwise compared and subsequently averaged. For testing of statistical differences, ScanMatch scores were entered to a two-factorial ANOVA for repeated measures, with type of contrast (imagewise, subjectwise) and time bin (1–10, 10–20, 20–30, 30–40) as within subject factors. In the ANOVA we compared ScanMatch scores of 27 participants and 60 paintings. Therefore, we performed 1.000 ANOVAs, each with a random selection of 27 out of 60 paintings. We found no reliable differences for type of contrast, since 60% of the tests revealed *p* > 0.05, but highly significant differences for time bin, *F*_(3, 78)_ = 64, *p* < 0.001. Furthermore, there was a significant interaction of type of contrast × time bin, *F*_(3, 78)_ > 0.9, with 87% of the tests revealing *p* < 0.05 (Figure 1C). The significant main effect for time bin was based on the larger ScanMatch scores of the first time bin compared to the subsequent time bins, indicating highest viewing similarity within the first 10 s. The interaction was qualified by larger ScanMatch scores for the picturewise analysis in the first time bin, while no differences were found for the subsequent time bins. Thus, the synchrony of spatial and temporal gaze behavior was highest across participants within the same painting but only during the first 10 s. The strongest drop in similarity can be found from the first to the second time bin, revealing that the most pronounced change in viewing behavior takes place within the first 20 s.

Finally, comparing similarity in scanpaths between the first and last 20 pictures per subject i.e., examining influences of time on task, revealed no reliable difference, *F*_(1, 26)_ < 1.

### Psychophysiological data

**Figure 2:**
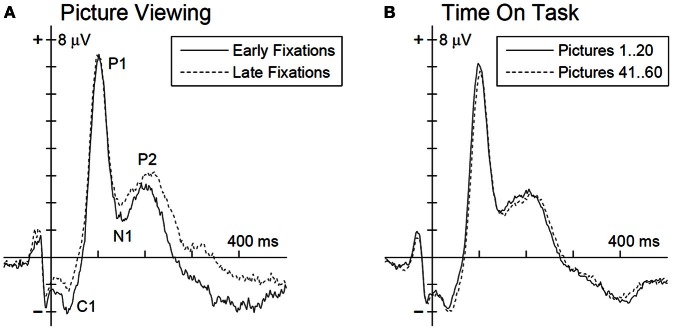
**Grand average EFRP for an occipito-parietal electrode cluster (PO3, POz, and PO4) for the analysis of picture viewing time (A) and time on task influences (B).** Ordinate axis denotes the fixation onset. EFRP components of interest are annotated in panel **(A)**.

The multivariate analysis, testing for EFRP differences between early and late time bin during picture viewing, revealed a significant main effect, *F*_(4, 23)_ = 4.73, *p* < 0.01. The univariate tests show for the C1, N1, and P2 components significant differences between early and late time bin. As illustrated in Figure 2A and listed in Table 1, C1 and N1 amplitudes were more negative during the first 10 s. The reverse pattern was observed for the P2: the amplitude was larger in the late time bin. No difference was found for P1 component.

**Table 1:** **Mean activity of EFRP components from early and late phases during picture viewing and the univariate test statistics**.

**EFRP component**	**Time window**		**Statistics**
	**Early**	**Late**	***F*_(1, 26)_**
C1	−1.53 (1.4)	−0.77 (1.35)	14.2^***^
P1	6.01 (2.98)	6.08 (3.4)	n.s.
N1	1.70 (2.16)	2.17 (2.38)	4.37^*^
P2	1.77 (1.66)	2.45 (2.16)	7.53^*^

* p < 0.05;

*** *p < 0.001; n.s. = p > 0.10*.

Furthermore, we compared power in the beta and theta frequency band for electrodes from a frontal-ROI between the early and late time bin. Multivariate testing revealed no differences in band-power as function of viewing time within a picture, *F*_(2, 25)_ = 3.01, *p* = 0.07.

For the analysis time on task effects, EFRPs of the first 20 and last 20 pictures in the experiment were matched (Figure 2B). The multivariate analysis revealed no time on task effect on the EFRP components, *F*_(4, 23)_ = 1.33, *p* = 0.29.

**Figure 3:**
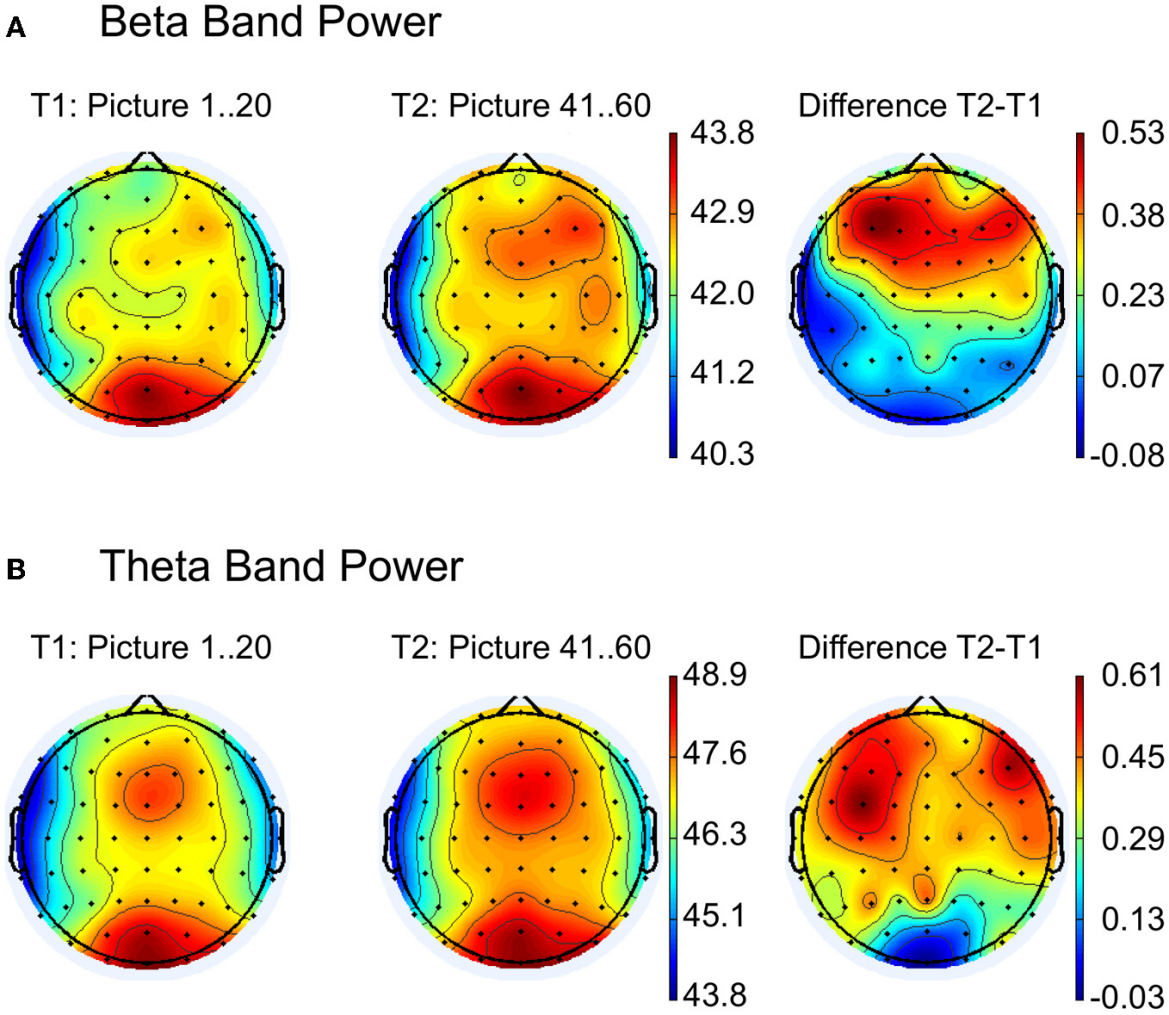
**Topographic maps for comparison between the first (T1) and the last (T2) 20 pictures for (A) beta and (B) theta band power.** The right column shows the difference maps T2 − T1. Strongest activity for beta and theta band is visible at the occipital electrode positions (see left and middle column), but a second activity pattern appears at frontal leads. As indicated in the difference maps (right column), main frequency differences between T2 and T1 occur at the frontal regions.

The topography of spectral beta and theta power over the scalp for EFRPs from the first and last 20 pictures of the experiment are illustrated in Figures 3A,B. The difference maps in Figure 3 indicate stronger beta and theta power over frontal regions during the last 20 images. Statistical testing (multivariate analysis) of the band power for the a priori defined frontal ROI revealed a significant difference between the first and last 20 pictures, *F*_(2, 25)_ = 12.24, *p* < 0.001. Univariate testing demonstrated higher beta activity (early: *M* = 42.9, *SD* = 3.46; late: *M* = 42.5, *SD* = 3.22), *F*_(1, 26)_ = 15.4, *p* < 0.001, as well as higher theta power (early: *M* = 48.0, *SD* = 3.4; late: *M* = 48.4, *SD* = 3.34), *F*_(1, 26)_ = 11.1, *p* < 0.001, for the last compared to the first 20 pictures.

## Discussion

We investigated behavioral and psychophysiological parameters during the free exploration of representational paintings in order to obtain further insights into the temporal dynamics of attentional control mechanisms. Electronic copies of paintings were shown for 40 s while eye movements and EEG were recorded simultaneously. We analyzed parameters of gaze behavior and fixation-related EEG-activity by comparing the initial 10 viewing seconds with the subsequent 30 s of each picture. We contrasted the same parameters in search for time on task effects by comparing gaze behavior and brain activity between the first and last 20 pictures of the experiment.

Analyses of gaze behavior revealed shortest fixation durations and largest saccade amplitudes during the first 10 s. Furthermore, the examination of viewing similarity indicated highest interindividual congruency during the initial 10 seconds of picture inspection. In contrast, comparing these parameters across the first 20 and last 20 pictures of the experiment revealed no changes.

The psychophysiological indicators also revealed particular differences. The ERFP components C1, N1, and P2 varied only during the 40 s of picture viewing but not between the first and last 20 pictures of the experiment. Larger negative amplitudes in C1 and N1 components were found during the initial 10 s compared to the subsequent exploration. In contrast, for P2, amplitudes were initially smaller. The analyses in the frequency domain of the EFRPs demonstrated changes only on the larger time scale. The frontal theta and frontal beta band power increased with time on task but remained stable throughout picture viewing.

During the initial 10 s of picture viewing, we observed shortest fixation duration and largest saccade amplitudes. This initial gaze behavior has already been reported (Antes, 1974; Unema et al., 2005) and was even suggested as an expression of bottom-up processing (Pannasch et al., 2008). Eye movement recordings have often been used to investigate influences of the given task (Yarbus, 1967), as well as saliency-driven bottom-up guidance (Underwood and Foulsham, 2006; Underwood et al., 2006). Massaro et al. (2012) explicitly investigated the relationship between bottom-up and top-down processes comparing task requirements and image features such as content and color. The most pronounced indicator for bottom-up influences was found for naturalistic paintings evidenced by shorter and more widespread fixations. Since about two-thirds of our stimulus material corresponds to the naturalistic category by Massaro et al. (2012), the initial short fixations and long saccades are likely to indicate bottom-up processing also in the present work. This seems furthermore supported by the fact that similarity is largest during the initial 10 s and drops subsequently. While this might be a valid interpretation at the first glance, it seems rather contradictory considering the fact that similarity was also highest when comparing the similarity subjectwise across images. Since all paintings are different, this early correspondence in spatial-temporal viewing behavior might rather be an expression of the central fixation bias (Tatler, 2007; Tatler et al., 2011). This interpretation is supported by the fact that a central fixation cross was shown before the image onset, i.e., each exploration started from the image center. How can we integrate an initial stronger bottom-up influence and the central fixation bias? It is known that in art, main figurative elements often appear in a central position (Locher et al., 2007; Tyler, 2007), thereby inducing intense scanning of these regions. The correspondence of viewing strategies was largest for the early exploration of the same picture by different participants. Under these circumstances, visual attention is similarly allocated which could be accounted best by bottom-up guidance to regions of highest saliency.

Such an interpretation is further supported by the modulation of the earliest EFRP activity. The amplitude of the C1 component was larger for the first 10-s time bin. It has been shown already, that the C1 arises from neural generators in the primary visual cortex (Di Russo et al., 2002). This brain region has also been proposed to create a saliency map via intracortical interactions (Li, 1999, 2002). Recently, by employing a masking design to analyze ERP and BOLD signal, Zhang et al. (2012) observed a relatively pure saliency signal. The authors observed that C1 amplitudes increased with saliency. To further support this line of argumentation, C1 was found to be not modulated by high vs. low attentional load (Fu et al., 2010). However, care has to be taken by adapting these results to the present work. Although we carefully selected the EFRPs for the two distinct phases further influencing factors could be possible in our free viewing experiment (for a recent discussion on C1, see Fu et al., 2012).

In agreement with numerous other studies, we observed a fixation duration increase as a function of the viewing time (Antes, 1974; Unema et al., 2005; Pannasch et al., 2008; Mills et al., 2011). Longer fixation duration has been related to more elaborated and detailed processing of fixational content (Loftus and Mackworth, 1978). It thus may be feasible to assume that the processing of information changes with inspection time toward a modus of deeper processing, possibly facilitated by knowledge acquired during the initial seconds of exploration. Functions of WM may play an essential role to enable such elaborated processing. Yet, in what order the information are selected depends strongly on individual characteristics, such as motivation, intention and goals and previous experience. These individual factors may strongly contribute to the decreasing consistency in eye movement patterns between subjects during late phases of image inspection.

Recent research has advocated the view that WM and selective attention are tightly interconnected phenomena (Awh and Jonides, 2001; Pratt et al., 2011; Gazzaley and Nobre, 2012). Electrophysiological research on this topic may thus help to understand the results obtained in our study. One finding in this domain is that the amplitude of the N1 component seems to correlate with the ability to direct selective attention and to react fast and appropriately to targets especially when WM load is high due to a secondary task (Rose et al., 2005). It was found that N1 amplitude decreased and distractibility increased as function of WM load. A similar explanation may be applied to our findings, where the N1 amplitude decreased as a function of 40 s of scene exploration. This may reflect an increase in demands on WM during inspection. Low WM load can be assumed after picture onset since new information is presented. With the ongoing inspection information about the scene, its objects and specific relations accumulates in WM. These pieces of information have to be stored but also compared and integrated with the prior knowledge from long term memory. Following this argumentation, N1 variation may be correlated with the changing WM demands during image exploration.

The P2 amplitude of the visual evoked potential has also been associated with states of selective attention. It was proposed that this component may express enhanced cognitive processing demands or processes of active inhibition, particularly in situations when expected targets and irrelevant stimuli appear simultaneously (Kotchoubey, 2006; Freunberger et al., 2007). An increase in P2 might thus either reflect stronger focusing on targets or higher demands to suppress irrelevant information which both are necessary during a state of focused attention. This inhibitory aspect is in particular apparent in experimental paradigms using distractor stimuli (Hickey et al., 2009). Since top-down control serves as a common neural mechanism for selective attention and WM (Gazzaley and Nobre, 2012), we assume that our findings for N1 and P2 illustrate a general bias toward top-down modulations across inspection time.

While the parameters of gaze behavior as well as the components of the EFRPs remained stable from the first to last 20 pictures of the experiment, we observed a pronounced increase in the frontal beta and theta power over that time. Along with this variation our subjects reported increased sleepiness and subjective strain with time on task. Similar results of increased frontal beta activity and subjective strain were previously reported for low bottom-up stimulation when sustained attention was required for an appropriate completion of the experimental task (Smit et al., 2004; Barbato et al., 2007; Fischer et al., 2008). Increased frontal theta activity was previously related to WM load (Gevins et al., 1997; Jensen and Tesche, 2002) and to sustained attention (Sauseng et al., 2007). According to Sauseng et al. (2007) it is possible to differentiate between the two effects: while sustained attention is expressed by higher frontal theta activity, memory processing can be identified by increased connectivity in theta activation between frontal and parietal regions. Considering this interpretation, our results of increased theta and beta activity together with the larger self-reported strain and sleepiness demonstrate indications of higher demands on sustained attention later during the experiment.

Taken together, our study revealed systematic variation in parameters of behavioral and psychophysiological measures which seems to indicate a general adaption of attentional mechanisms in the time course of naturalistic image exploration. Early during inspection, we found a pattern that suggests a stronger influence of bottom-up control on attentional selection and processing. This early period is followed by a change that suggests an increasing impact of top-down controlled attentional processes. This, however, is a rather coarse interpretation of the current observations since dynamics and competition between these two attentional mechanisms may be much more vital on a finer time scale. While our findings reveal a shifting balance between bottom-up and top-down attentional guidance, it remains open which of the two mechanisms plays the dominating role to direct attention and control eye movements. Furthermore, it cannot be clarified how the interplay between the attentional processes exactly changes. As it looks from the present results so far, during visual exploration bottom-up activity decreases while at the same time the top-down influence increases. However, other interactions between both mechanisms are conceivable: Bottom-up activity remains stable but only top-down influences increase or vice versa. Further research should answer this question by explicitly testing these hypotheses.

We did attempt for the first time to explore aspects of the dynamic interaction between different attentional mechanisms and their neuronal correlates under relatively naturalistic conditions. Although we found a dynamic interaction between the discussed attentional mechanisms, understanding the precise nature of the interaction needs further investigation. Furthermore, our approach was grounded on the concepts of bottom-up, top-down, and sustained attention, alternative approaches for the explanation of naturalistic viewing should also be considered in further studies (Hochstein and Ahissar, 2002). Finally, more clarification is needed on how WM load can influence the EFRPs components during free exploration.

### Conflict of interest statement

The authors declare that the research was conducted in the absence of any commercial or financial relationships that could be construed as a potential conflict of interest.
